# The role of advanced glycation end products in fracture risk assessment in postmenopausal type 2 diabetic patients

**DOI:** 10.3389/fendo.2022.1013397

**Published:** 2022-12-12

**Authors:** Liu Gao, Chang Liu, Pan Hu, Na Wang, Xiaoxue Bao, Bin Wang, Ke Wang, Yukun Li, Peng Xue

**Affiliations:** ^1^ Department of Endocrinology, The Third Hospital of Hebei Medical University, Shijiazhuang, China; ^2^ Key Laboratory of Orthopedic Biomechanics of Hebei Province, The Third Hospital of Hebei Medical University, Shijiazhuang, China; ^3^ Trauma Medicine Center, Peking University People’s Hospital, Beijing, China; ^4^ National Center for Trauma Medicine, Peking University People's Hospital, Beijing, China

**Keywords:** type 2 diabetes mellitus, osteoporosis, fracture risk, FRAX, advanced glycation end products

## Abstract

**Objective:**

The objective of this study was to analyze the quantitative association between advanced glycation end products (AGEs) and adjusted FRAX by rheumatoid arthritis (FRAX-RA) in postmenopausal type 2 diabetic (T2D) patients. The optimal cutoff value of AGEs was also explored, which was aimed at demonstrating the potential value of AGEs on evaluating osteoporotic fracture risk in postmenopausal T2D patients.

**Methods:**

We conducted a cross-sectional study including 366 postmenopausal participants (180 T2D patients [DM group] and 186 non-T2D individuals [NDM group]). All the subjects in each group were divided into three subgroups according to BMD. Physical examination, dual-energy x-ray absorptiometry (DXA), and serum indicators (including serum AGEs, glycemic parameters, bone turnover markers and inflammation factors) were examined. The relationship between FRAX-RA, serum laboratory variables, and AGEs were explored. The optimal cutoff value of AGEs to predict the risk of osteoporotic fracture was also investigated.

**Results:**

Adjusting the FRAX values with rheumatoid arthritis (RA) of T2D patients reached a significantly increased MOF-RA and an increasing trend of HF-RA. AGEs level was higher in the DM group compared to the NDMs, and was positively correlated with MOF-RA (r=0.682, *P<0.001*) and HF-RA (r=0.677, *P*<0.001). The receiver operating characteristic curve analysis revealed that the area under the curve was 0.804 (*P*<0.001), and the optimal AGEs cut-off value was 4.156mmol/L. Subgroup analysis for T2D patients revealed an increase in TGF-β, IL-6 and SCTX in the osteoporosis group, while a decreased PINP in the osteoporosis group compared to the other two subgroups. AGEs were positively associated with FBG, HbA1c, HOMA-IR, S-CTX, IL-6 and TGF-β in T2D patients, and negatively associated with PINP.

**Conclusions:**

RA-adjusted FRAX is a relevant clinical tool in evaluating fracture risk of postmenopausal T2D patients. Our study analyzed the relationship between AGEs and FRAX-RA, and explored the threshold value of AGEs for predicting fracture risk in postmenopausal T2D patients. AGEs were also associated with serum bone turnover markers and inflammation factors, indicating that the increasing level of AGEs in postmenopausal T2D patients accelerated the expression of inflammatory factors, which led to bone metabolism disorders and a higher risk of osteoporotic fractures.

## Introduction

Osteoporosis is prevalent in type 2 diabetes mellitus (T2D) postmenopausal patients, which affects human health, life quality and increases the socioeconomic burden (1). T2D patients have bone mineral density (BMD) that is either unchanged or slightly higher than normal, but they exhibit skeletal fragility independent of BMD (2, 3), even after accounting for some factors (such as body mass index [BMI] and falls) (4, 5), which indicates patients with T2D have a higher fracture risk due to bone fragility independent of BMD. Besides, Diabetes status was associated with low muscle mass and low muscle strength, and the association depended on BMI (6). The concomitance of sarcopenia and osteoporosis which was so-called “osteosarcopenia”, may lead to an increase in fracture risk of T2D than the non-diabetic ones (7). Older adults with osteosarcopenia have to be regarded as the most at-risk population for fractures (8). Thus, the unadjusted fracture risk assessment tool (FRAX) mostly depends on dual-energy x-ray absorptiometry (DXA) detection could also underestimate the fracture risk in T2D patients (9, 10).

Approximately 70% of bone strength is determined by BMD, while collagen fiber composition depends on bone tissue’s tensile strength and ductility. Collagen molecular crosslinking can be divided into beneficial enzyme-catalyzed immature bivalent cross-linking and mature trivalent crosslinking, and unfavorable non-enzyme-catalyzed crosslinking, such as advanced glycation end products (AGEs). AGEs are the spontaneous reaction products between extracellular sugars and amino acid residues on collagen fibers (11). The accumulation of AGEs in the bone can reduce skeletal hardness biomechanical properties (12). Previous studies showed a significantly increased AGEs level in T2D patients (13, 14), which was related to low bone quality and high fracture risk in postmenopausal women (15). Meanwhile, the accumulation of AGEs is associated with impaired bone microarchitecture. It has been reported that AGEs bone content correlated with worse bone microarchitecture in trabecular, including lower volumetric BMD, bone volume fraction, and increased separation/spacing (16). Bone microarchitecture could be regarded as an independent predictor of fracture risk (17). Although the FRAX includes some diseases related to osteoporosis, other risk factors were not accounted for, such as falls, the duration and dosage of glucocorticoids, the etiology and type of diabetes, or other secondary osteoporosis. FRAX base on BMD may not always accurately predict the fracture risk of T2D patients. Therefore, we speculate that abnormal cross-linking of collagen molecules may be an important factor contributing to impaired bone quality and increased skeletal fragility, which increased the fracture risk in postmenopausal T2D patients.

In this study, we adopted a method previously reported by both Hu et al. and Leslie et al. (18, 19), rheumatoid arthritis (RA) was selected as an analogous variable of T2D to obtain the FRAX predictive value for fracture risk. Thus, the objective of this study was to analyze the quantitative association between AGEs and adjusted FRAX by RA (FRAX-RA) in postmenopausal T2D patients. The optimal cutoff value of AGEs was also explored, which was aimed at demonstrating the potential influence of AGEs on osteoporotic fracture risk in postmenopausal T2D patients. Moreover, we tried to use HR-pQCT to verify the status of bone microstructure of T2DM patients in “High-AGEs” or “Low-AGEs” group defined by its cut off value in a small-size sample.

## Materials and methods

### Subject recruitment

We collected 180 postmenopausal T2D patients (DM group) and 186 healthy individuals (NDM group) who were recruited from the Endocrinology Department of the Third Hospital of Hebei Medical University from October 2019 to May 2020. Each cohort was divided into three subgroups (non-diabetic subjects with normal BMD [Control], non-diabetic subjects with osteopenia [OPN], non-diabetic subjects with osteoporosis [OP], diabetic patients with normal BMD[DMN], diabetic patients with osteopenia [DMOPN], diabetic patients with osteoporosis [DMOP]) according to BMD. All subjects submitted written informed consent prior to participating in this study, which was authorized by the Third Hospital of Hebei Medical University’s ethical committee.

### Inclusion and exclusion criteria

The subjects were chosen based on the following criteria: 1) All subjects were aged between 45 and 80, natural menopause for more than 3 years or menopause caused by surgery (operating time after 40 years old); 2) the WHO’s (1999) diabetes criteria: diabetic symptoms (polydipsia, polyuria, polyphagia, weight loss) + blood glucose level at any time ≥ 11.1mmol/L or fasting glucose ≥ 7.0mmol/L or 2hours postprandial glucose≥11.1mmol/L. Type 1 diabetes mellitus were excluded from this study; 3) the WHO’s osteoporosis criteria: the diagnosis of osteoporosis in postmenopausal women is based on the T value. T value ≥ -1SD was normal bone mineral density, -1SD < T value < -2.5SD was osteopenia; T value ≤ -2.5SD was osteoporosis.

Subjects with these conditions were excluded: severe heart, liver, and kidney disease, thyroid and parathyroid disease, autoimmune disease, rheumatism, long-term use of hormones and thiazide diuretics, use of antidiabetic drugs that may affect bone metabolism for more than three months (metformin, thiazolidinediones, glucagon-like peptide-1 receptor agonist, sodium-glucose cotransporter 2 inhibitor), long-term stay in bed or chronic smoking (smoking for more than 15 years, averaging more than 15 cigarettes a day), BMI is less than 20kg/m^2^.

### Laboratory assessment

We collected data from all subjects (including age, menopausal age, weight, and height), measured serum concentrations of fasting plasma glucose (FPG), glycosylated hemoglobin (HbA1c) and fasting insulin (FIns) by using standard laboratory techniques, measured serum AGEs, insulin, 25-hydroxyvitamin D3 (25-OHD3), procollagen type I N-peptide (PINP), serum C-terminal telopeptide of type I collagen (S-CTX) by the enzyme-linked immunosorbent assay (ELISA) kit (Cusabio, Wuhan, China), measured serum concentrations of insulin, Interleukin-1β (IL-1β), IL-6, tumor necrosis factor-α (TNF-α) and transforming growth factor-β (TGF-β) by the ELISA kit (Excellbio, shanghai, China). BMI was determined using the following equation: BMI=Weight/Height^2^ (kg/m^2^). The following formula was used to calculate the insulin resistance index (HOMA-IR): HOMA-IR = FPG * FIns/22.5.

### BMD assessment

We evaluated the level of areal BMDs at the lumbar spine (LS, L2-L4), proximal femur (femoral neck and total hip) for each individual using a DXA device (Hologic, USA). The measurements were all taken by the same technician to ensure consistent and reliable results, and the CVs were 1.73% across the board.

### Fracture risk assessment tool

The predicted 10-year risk of major and hip osteoporotic fractures was determined using the Asian-China Assessment System (https://www.sheffield.ac.uk/FRAX/tool.aspx?country=2). The FRAX algorithm includes risk factors of age, gender, height, weight, previous fracture history, parents’ history of fragility fractures, smoking status, long-term corticosteroid use, RA history, daily alcohol consumption, secondary OP, and femoral neck bone density. The history of RA was replaced in the algorithm for the current study to calculate FRAX-RA.

### Bone microarchitectural measurements

HR-pQCT was used to verify the status of bone microstructure of T2DM patients in both “High-AGEs” (AGEs>4.156mmol/L) or “Low-AGEs” (AGEs<4.156mmol/L) group defined by its cut off value. We chose 14 subjects aged 50-60yr without fracture history (8 in High-AGEs group and 6 in Low-AGEs group) underwent HR-pQCT of the nondominant distal radius and tibia (Xtreme CT II; Scanco Medical AG, Bassersdorf, Switzerland) according to the manufacturer’s standard *in vivo* acquisition protocol (68 kVp, 1470 μA, matrix size of 2304×2304) (20). The reference line was placed at the endplates of the distal radius and tibia in all tested participants. The scan region was 10.2mm in length, and was fixed starting at 9.0 mm and 22.0 mm proximal to the reference lines of the radius and tibia respectively. The measured parameters were as follows: cortical thickness (Ct.Th, mm); cortical porosity (Ct.Po, %); trabecular bone volume fraction (Tb.BV/TV, %), number (Tb.N, 1/mm), thickness (Tb.Th, mm) and separation (Tb.Sp, mm). The measurements were all taken by the same technician to ensure consistent and reliable results.

### Statistical analyses

All statistical analysis was performed using SPSS version 21.0. We used the Kolmogorov-Smirnov test to confirm the normal distribution of variables for each group. The median (interquartile range) was used to express data for non-continuous variables, whereas the mean ± SD was used to express data for continuous variables. The Student’s T-Test is utilized to compare 2 groups that adhere to the normal distribution and uniform variance, and the Wilcoxon test is used to compare 2 groups that do not obey the normal distribution. We used the ANOVA or Friedman test to compare the quantitative variables among groups. Pearson or Spearman correlation tests were used to determine relationships between variables. In order to determine or assess the best AGE cutoff value for predicting or evaluating the risk of osteoporotic fracture, receiver operating characteristic (ROC) curves were used. Maximum sensitivity and specificity for fracture risk are achieved by the cut-off value. Estimating the area under the curve was served to evaluate the test’s discriminatory ability. A difference with a P value of 0.05 or lower is considered statistically significant for all statistical tests.

## Results

### Baseline features of the subjects

The general characteristics of the subjects are displayed in **Table 1**. T2D patients had higher BMI compared to non-diabetics (*P*=0.034), while the two groups were comparable in age and menopause duration. Analysis of subgroups indicates that BMI in DMOP group was considerably lower than in DMN and DMOPN groups (*P*<0.05), and OP group had significantly lower BMI than the Control and OPN groups (*P*<0.05).

**Table 1:** Baseline characteristics of the study population.

	NDM					DM				
	Total (n=186)	Control (n=58)	OPN (n=63)	OP (n=65)	*P Value*	Total (n=180)	DMN (n=52)	DMOPN (n=60)	DMOP (n=68)	*P Value*
Age (years)	63.978 ± 9.234	63.621 ± 8.626	63.444 ± 9.193	64.815 ± 9.861	0.662	65.000 ± 8.574	64.423 ± 8.696	64.383 ± 8.015	65.985 ± 8.983	0.488
Menopausal duration (years)	14.000 (13.000)	13.000 (11.000)	13.000 (11.000)	16.000 (17.000)	0.529	15.000 (10.750)	15.000 (15.500)	14.000 (9.000)	15.000 (12.000)	0.780
T2D duration (years)	–	–	–	–	–	10.000 (9.750)	10.000 (8.750)	11.000 (12.000)	8.000 (13.000)	0.169
Fracture history, n (%)	17/186 (9.140%)	2/58 (3.448%)	2/63 (3.175%)	13/65 (20.000%)^△※^	<0.001	17/180 (9.444%)	2/52 (3.846%)	3/60 (5.000%)	16/68 (23.529%)^△※^	<0.001
BMI (kg/m^2^)	24.361 ± 3.264	25.437 ± 3.218	24.587 ± 3.252	23.181 ± 2.967^△※^	<0.001	25.422 ± 4.353	25.421 ± 3.358	26.943 ± 4.968	24.082 ± 4.050^*#^	<0.001
MOF	3.750(2.700)	3.050(1.300)	3.500(2.300)	6.100(5.100)^△※^	<0.001	3.500(2.300)	2.700(1.750)	3.100(1.100)	4.950(3.450)^*#^	<0.001
HF	0.900(1.825)	0.450(0.600)	0.800(1.100)	2.500(4.000)^△※^	<0.001	0.900(1.200)	0.400(0.775)	0.600(0.900)	1.700(2.000)^*#^	<0.001
MOF-RA	–	–	–	–	–	4.650(3.700)	3.500(2.250)	4.050(2.750)	6.700(3.950)^*#^	<0.001
HF-RA	–	–	–	–	–	1.450(2.100)	0.800(1.275)	0.900(1.600)	2.750(3.100)^*#^	<0.001

^△^P < 0.05 compared to Control group, ^※^P < 0.05 compared to OPN group, ^*^P < 0.05 compared to DMN group, ^#^P < 0.05 compared to DMOPN group.

Control, non-diabetic subjects with normal BMD; OPN, non-diabetic subjects with osteopenia; OP, non-diabetic subjects with osteoporosis; DMN, diabetic patients with normal BMD; DMOPN, diabetic patients with osteopenia; DMOP, diabetic patients with osteoporosis; MOF, major osteoporotic fractures; HF, hip osteoporotic fractures; MOF-RA, MOF adjusted by rheumatoid arthritis; HF-RA, HF adjusted by rheumatoid arthritis.

### BMD, FRAX, and RA-FRAX comparation among DM and NDM groups

**Figure 1:**
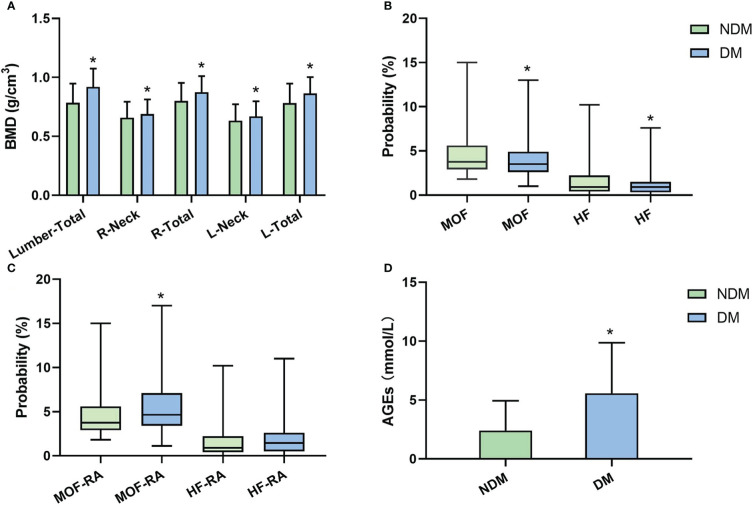
BMD, FRAX values (without correction), FRAX values (corrected by rheumatoid arthritis [RA]), and AGEs level between NDM and DM groups. **(A)** BMD comparation in each area; **(B)** major osteoporotic fractures (MOF) and hip osteoporotic fractures (HF) comparation; **(C)** Adjusted MOF by RA (MOF-RA) and adjusted MOF by RA (HF-RA) comparation; **(D)** AGEs comparation. (*P<0.05 compared to the NDM group).

DM group had substantially higher BMD than the non-diabetics (*P*<0.05), as shown in **Figure 1A**. The probabilities of major osteoporotic fractures (MOF) and hip osteoporotic fractures (HF) in T2D patients were lower than the non-diabetics (*P*<0.05, **Figure 1B**). Then, in order to obtain MOF-RA and HF-RA, we altered the FRAX values of T2D patients by choosing RA as an analogous variable. A significant increase of MOF-RA in DM group was found than the NDM group (*P*<0.05), while DM group tends to have higher HF-RA than NDM group (**Figure 1C**).

### AGEs level comparison between DM and NDM groups

**Figure 2:**
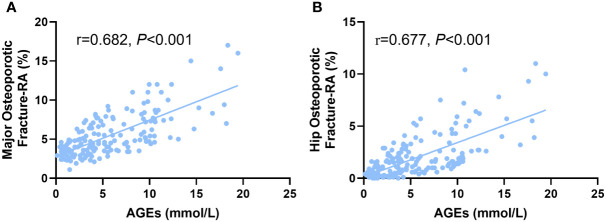
The correlation of AGEs with **(A)** major osteoporotic fractures and **(B)** hip osteoporotic fractures adjusted by rheumatoid arthritis (RA) in postmenopausal T2D patients.

In comparison to non-diabetics, we found that DM patients had considerably higher AGEs levels (*P*<0.05, **Figure 1D**). According to Pearson correlation analysis, AGEs level was positively correlated with MOF-RA (r=0.682, *P<*0.001) (**Figure 2A**) and HF-RA (r=0.677, *P<*0.001) (**Figure 2B**).

### Evaluation of the AGEs optimal cutoff value to predict osteoporotic fracture risk

**Figure 3:**
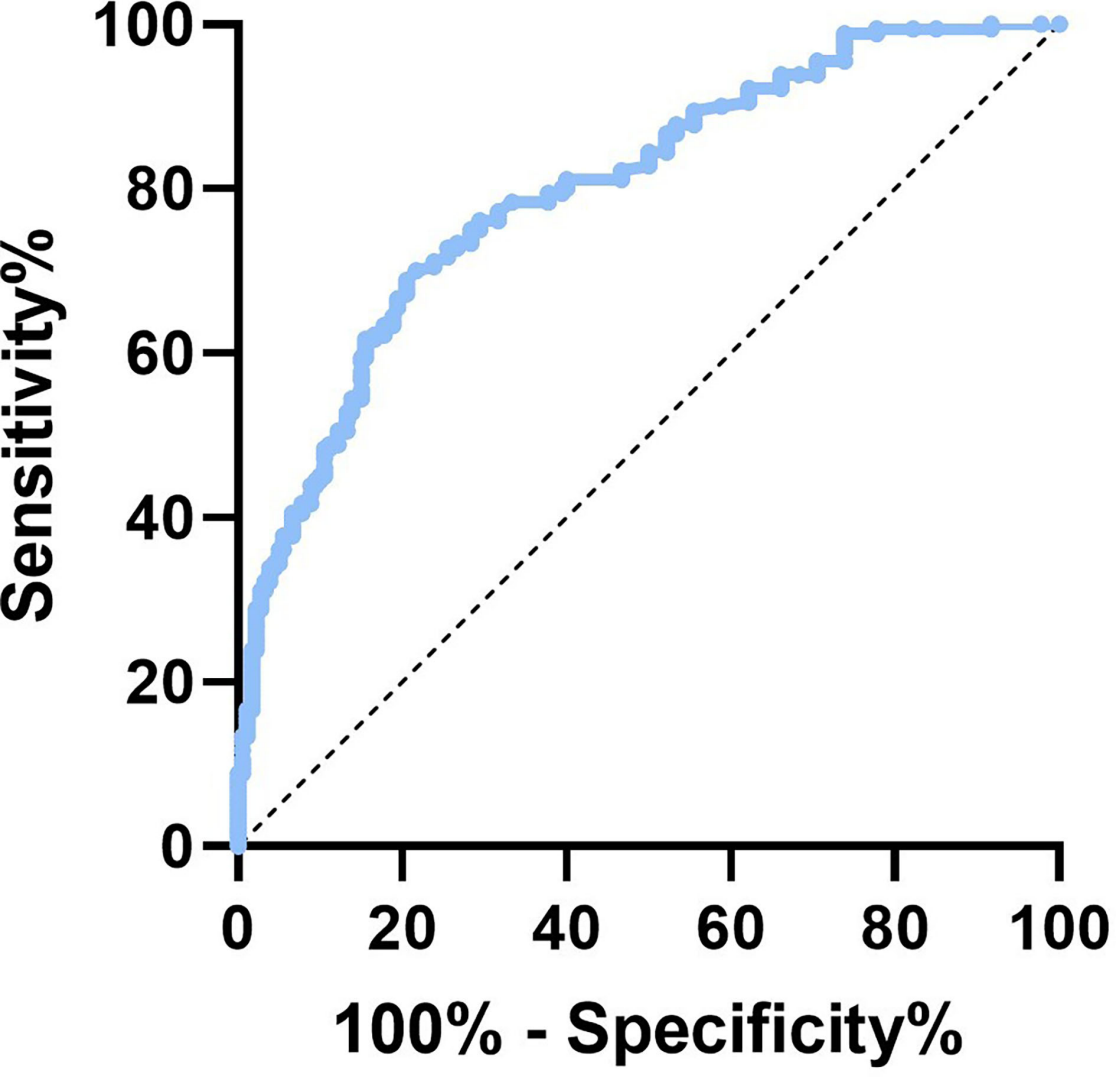
The ROC curve of AGEs in predicting fracture risk of postmenopausal T2D patients.

To determine the ideal AGE cut-off value for evaluating fracture risk in postmenopausal T2D patients, the ROC curve was used. As shown in **Figure 3**, the area under ROC curve (AUC) was recorded as 0.804 (95% confidence interval [CI]:0.749-0.858, *P*<0.001), and the optimal AGEs cut-off value leading to a high fracture risk was 4.156mmol/L. This suggests postmenopausal T2D patients have an increased risk of fracture when AGEs level is higher than 4.156mmol/L. We then tried to verify our AGEs cut-off value by measuring bone microstructure in T2D postmenopausal women. A total of 14 subjects aged 50-60yr without fracture history underwent HR-pQCT examination in nondominant distal radius and tibia, 8 in High-AGEs group and 6 in Low-AGEs group. The Ct.Po was increased in the High-AGEs group than the Low-AGEs group at tibia. And the results of radius were consistent with tibia (*P*<0.05, **Supplementary Figures 1D**, **2D**). No difference was found in Ct.Th and trabecular parameters (Tb.BV/TV, Tb.N, Tb.Th, and Tb.Sp) between these two groups in both tibia and radius (**Supplementary Figures 1A–C, E–H**, **2A–C, E–H**).

### Glucose parameters, bone turnover markers, and inflammation factors comparation among DMN, DMOPN and DMOP groups

First, no differences were found when comparing FBG, HbA1c, insulin, and HOMA-IR among the three groups (*P*>0.05, **Table 2**). Then we compared bone turnover markers among the three groups. Results revealed that the DMOP group had lower levels of PINP and 25-OHD3 than the DMN and DMOPN groups (*P*<0.05, **Table 2**), while an increase of S-CTX in DMOP group than the other two groups. Next, the comparison of inflammation factors showed that DMOP patients had higher IL-6 and TGF-β levels compared to both DMN and DMOPN groups (*P*<0.05, **Table 2**). However, in terms of IL-1β and TNF-α, there were no noticeable differences among the three groups (*P*>0.05, **Table 2**).

**Table 2:** Glucose parameters, bone turnover markers, and inflammation factors comparation among DMN, DMOPN and DMOP groups.

	Glucose parameters				Bone turnover markers			Inflammation factors			
	FBG (mmol/L)	HbA1c (%)	Insulin (mU/L)	HOMA-IR	25-OHD_3_ (μg/L)	PINP (pg/ml)	S-CTX (ng/ml)	IL-1β (pg/ml)	IL-6 (pg/ml)	TNF-α (pg/ml)	TGF-β (pg/ml)
DMN (n=52)	8.450 (3.250)	8.792 ± 2.462	16.070 (11.455)	5.889 (5.220)	3723.175 ± 940.977	3822.100 (3654.725)	5.028 (4.518)	4.409 (4.070)	3.185 (7.021)	34.493 (42.574)	24470.560 ± 7809.782
DMOPN (n=60)	8.300 (4.725)	8.875 ± 2.255	15.056 (11.017)	5.249 (4.229)	2830.517 ± 794.228	3744.500 (1314.45)	5.883 (3.985)	6.394 (5.058)	3.152 (5.750)	37.802 (34.771)	27949.802 ± 8559.8723
DMOP (n=68)	8.150 (3.600)	8.893 ± 2.041	15.557 (12.046)	5.125 (5.084)	2819.125 ± 883.256	3068.900 (1298.325)^*#^	7.527 (7.209)^*#^	6.233 (1.935)	4.535 (6.575) ^*#^	40.729 (57.955)	33206.162 ± 7112.012^*#^
*P Value*	0.873	0.968	0.991	0.930	0.449	<0.001	<0.001	0.165	<0.001	0.418	<0.001

^*^P < 0.05 compared to DMN group, ^#^P < 0.05 compared to DMOPN group.

DMN, diabetic patients with normal BMD; DMOPN, diabetic patients with osteopenia; DMOP, diabetic patients with osteoporosis.

### Correlations of glycemic parameters, bone turnover markers, and inflammatory factors with AGEs among T2D patients

We used Spearman or Pearson correlation to analyze the relationship among glycemic parameters, bone turnover markers, inflammatory factors, and AGEs in postmenopausal T2D patients. As shown in **Table 3**, AGEs were positively correlated with FBG, HbA1c, and HOMA-IR levels (r=0.323, r=0.191, r=0.190 respectively, *P*<0.05). No linear correlations were found between AGEs and insulin. Besides, AGEs were negatively correlated with PINP (r=-0.161, *P*<0.05) and positively correlated with S-CTX (r=0.167, *P*<0.05), while no correlation was found between AGEs and 25-OHD3. Serum levels of AGEs were found to be significantly positively correlated with IL-6 and TGF-β (r=0.417, r=0.580 respectively, *P*<0.05), but no linear correlation was found between IL-1β, TNF-α and AGEs.

**Table 3:** Correlations of glycemic parameters, bone turnover markers, and inflammatory factors with AGEs among postmenopausal type 2 diabetic patients.

		Glucose parameters				Bone turnover markers			Inflammation factors			
		FBG(mmol/L)	HbA1c (%)	Insulin (mU/L)	HOMA-IR	25-OHD_3_ (μg/L)	PINP (pg/ml)	S-CTX (ng/ml)	IL-1β (pg/ml)	IL-6 (pg/ml)	TNF-α (pg/ml)	TGF-β (pg/ml)
AGEs	*r*	0.323	0.191	-0.025	0.190	0.044	-0.161	0.167	0.259	0.417	0.046	0.580
	*P*	<0.001	0.010	0.736	0.011	0.559	0.031	0.025	0.073	<0.001	0.097	<0.001

## Discussion

Osteoporosis is a frequent metabolic bone disease. Moreover, diabetic patients with osteoporosis would have a greater overall disease burden. Even after adjusting for BMD, BMI, visual impairment and falls, T2D individuals have a higher risk of fragility fractures (21). However, previous studies showed that individuals with T2D show unaltered (22, 23) or paradoxically increased (24, 25) BMD. Our results also showed that BMDs in DM group was significantly higher than the non-diabetics in postmenopausal women, which may partly be due to higher BMI. Therefore, diabetes-related changes in bone metabolism or biochemistry may be independent of other changes in bone microstructure and tissue properties other than BMD (26). Despite the fact that BMD understates the risk of fracture in diabetic patients, it remains to be the gold standard for evaluating bones in this population due to its high accessibility and low cost (27–29).

The most popular tool for assessing fracture risk is FRAX, and it can be used to calculate an individual’s 10-year risk of hip and severe osteoporotic fracture (30). Recent research indicates that T2D considerably raises fracture risk independent of other risk factors (31, 32). However, T2D is not one of the clinical risk variables in the FRAX algorithm. To increase the performance of FRAX in patients with T2D, it is advised to input RA to represent the condition of diabetes (18, 33). In the present study, we used a conventional BMD-based FRAX score to analyze the incidence of MOF and HF in all subjects and found both MOF and HF were significantly lower in DM patients. We subsequently selected RA as the equivalent variable of T2D based on the prior work to increase the precision of FRAX in the fracture risk evaluation of T2D patients (18), and found DM group had a significant increase in MOF-RA and a trend of higher HF-RA than NDM group. This result indicates that adjusting for RA when calculating FRAX may reflect the fracture risk of T2D patients more realistically.

After adjusting by RA, the FRAX score was numerically closer to the realistically fracture risk in T2D patients, but it could not explain the pathogenesis of the increased fracture risk in T2D individuals. Fractures are influenced by a complicated pathophysiological interplay between T2D parameters including a prolonged illness duration (34), diabetic complications, poor glycemic control (35), insulin resistance (36), and the use of insulin or oral antidiabetic medication (37, 38). It is yet unknown how deteriorating glycemic control might alter the characteristics of bone tissue. Hypothesized mechanisms include impaired bone remodeling, bone microvascular insufficiency, alterations in endocrine function, and accumulation of AGEs (21). It’s worth noting that in a prolonged hyperglycinemia state, glucose reacts with proteins to form AGEs, which may degrade bone tissue properties (39–42). The interaction of AGEs with the receptor (RAGE) on osteoblastic lineage cells results in decreased enzymatic collagen maturity, altered collagen fibrils profile, and further disrupts the mineralization process (43, 44). Additionally, the accumulation of AGEs leads to a promotion of inflammation and oxidative stress, which increases the differentiation and activation of osteoclasts (45) and induces osteoblast apoptosis (46, 47). This process also contributes to a low bone turnover state (48, 49). Our result showed that the elevated AGEs level was positively correlated with MOF-RA and HF-RA in postmenopausal women with T2D, indicating AGEs levels are strongly associated with fracture risk in T2D patients. The optimal AGEs cut-off value leading to a high fracture risk was 4.156mmol/L, which suggests postmenopausal T2D patients have an increased fracture risk when the AGEs level is higher than 4.156mmol/L. Previous studies indicated that the impaired bone microarchitecture has a considerable influence on bone strength and is essential in fracture initiation and propagation (50, 51). A meta-analysis reported the increase of cortical porosity is relevant to bone quality decline and increased fracture risk. It was also proved that cancellous bone preferentially accumulates AGEs relative to cortical bone (52). We thus verify the status of bone microstructure of T2DM patients in both High-AGEs or Low-AGEs group defined by its cut off value. The result showed the cortical porosity was increased in the High-AGEs group than the Low-AGEs group at tibia. No difference was found in cortical thickness and trabecular parameters between these two groups in both tibia and radius. These results were consistent with previous studies that AGEs bone content correlated with worse bone microarchitecture (16). However, Hunt et al. observed the trend of higher BV/TV values and greater mineral content in the T2D specimens which increased the bone strength (11). We speculated that the difference was because of Hunt et al. only analyzed cancellous bone structure, and their subjects was male T2D patients, which was quite different from us. Therefore, we suggest that the AGEs level as a correction factor that could improve the capacity of FRAX algorithm to predict fracture risk in T2D postmenopausal women.

Serum bone turnover markers can be used to assess bone loss or formation more sensitive than BMD (53–55). Previous studies demonstrated reduced bone resorption and formation in T2D individuals (56–58), suggesting that hyperglycemia and AGEs crosslinking may impair the function of osteoblasts and osteoclasts, thereby inhibiting bone formation and promoting bone resorption. Correlation analysis in our study also confirmed the AGEs level was positively correlated with glycemic parameters including FBG, HbA1c, HOMA-IR, bone resorption marker S-CTX, and negatively correlated with bone formation marker PINP in postmenopausal T2D patients. These findings imply that deteriorating glycemic control may contribute to the accumulation of AGEs, which interfere with normal osteoblast function and impair osteoblast development. AGEs may also reduce bone resorption by suppressing osteoclastic differentiation as well as changing the structural integrity of matrix proteins.

Patients with T2D have higher levels of AGEs due to hyperglycemia, which can also increase the production of inflammatory cytokines and reactive oxygen species, setting off a vicious cycle of chronic inflammation and bone resorption (59). Activating of RAGE in both osteoclasts (60, 61) and osteoblasts (46, 62) could induce up-regulation of pro-inflammatory cytokines such as IL-1β, IL-6, and TNF-α, which could directly affect bone homeostasis (63, 64). Accumulating evidence indicates that the TGF-β also plays an important role in the osteogenic progress affected by AGEs, especially biologically potent AGE2 and AGE3 (65, 66). Yamaguchi et al. conducted a series of studies and found that AGE2 and AGE3 suppressed stomal ST2 cell growth, differentiation, and mineralization, as well as increased apoptosis of osteoblastic cells by up-regulating TGF-β (67–69). As was shown in a clinical study, T2D patients have increased serum levels of IL-6, TGF-β, and TNF-α (70). We also found elevated levels of IL-6 and TGF-β in postmenopausal T2D patients, both of which positively correlated with AGEs levels. Thus, we hypothesized that the bone fragility and increased fracture risk of T2D patients may be due to AGE-induced IL-6 and TGF-β related inflammatory response. At present, the related mechanism of IL-6 and TGF-β on bone collagen abnormal cross-linking is still incomplete, and further research is needed.

In conclusion, both DXA and FRAX scores underestimated the accurate fracture risk in T2D patients. RA-adjusted FRAX is an efficient clinical tool for determining the risk of fracture in postmenopausal T2D patients. AGEs were also associated with serum bone turnover markers and inflammation factors, indicating that the increasing level of AGEs in postmenopausal T2D patients accelerated the expression of inflammatory factors, which led to bone metabolism disorders and a higher risk of osteoporotic fractures.

## Data availability statement

The original contributions presented in the study are included in the article/**Supplementary Material**. Further inquiries can be directed to the corresponding authors.

## Ethics statement

The studies involving human participants were reviewed and approved by Ethics Committee of the Third Hospital of Hebei Medical University. The patients/participants provided their written informed consent to participate in this study.

## Author contributions

PX, LG, CL and PH designed the research. LG, CL, PH, NW and XB organized the database. BW and KW performed the statistical analysis. PX, LG, CL and PH wrote the first draft of the manuscript. PX, YL and LG thoroughly revised the manuscript. All authors contributed to manuscript revision and approved the submitted version.
