# Supplementary material for: The role of advanced glycation end products in fracture risk assessment in postmenopausal type 2 diabetic patients
Source: Front Endocrinol (Lausanne). 2022 Dec 12;13:1013397. doi: 10.3389/fendo.2022.1013397 (PMC9790927; doi:10.3389/fendo.2022.1013397)
Supplement: Supplementary file 1 [file DataSheet_1.pdf]

Supplementary Material

1 Supplementary Figures

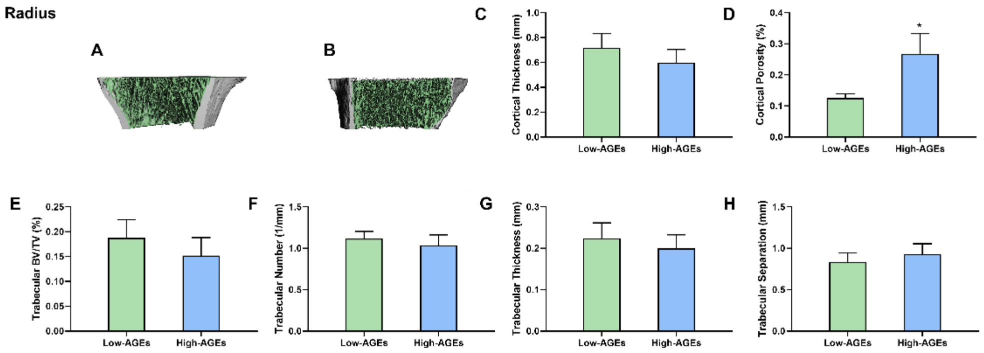

Supplementary Figure 1. Radius HR-pQCT images and parameters in Low-AGEs and High-AGEs groups of T2D postmenopausal women without history of fractures. (**P*<0.05 compared to Low-AGEs group)

A-B, Two representative radius HR-pQCT images from subjects in Low-AGEs group (A) and High-AGEs group (B) respectively; (C) cortical thickness; (D) cortical porosity; (E) trabecular bone volume fraction (BV/TV); (F) trabecular number; (G) trabecular thickness; (H) trabecular separation.

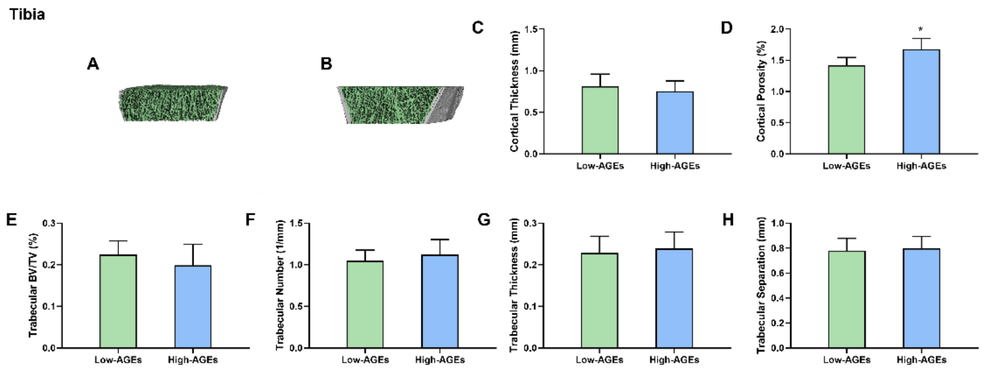

Supplementary Figure 2. Tibia HR-pQCT images and parameters in Low-AGEs and High-AGEs groups of T2D postmenopausal women without history of fractures. (**P*<0.05 compared to Low-AGEs group)

A-B, Two representative tibia HR-pQCT images from subjects in Low-AGEs group (A) and High-AGEs group (B) respectively; (C) cortical thickness; (D) cortical porosity; (E) trabecular bone volume fraction (BV/TV); (F) trabecular number; (G) trabecular thickness; (H) trabecular separation.
